# MR imaging to detect myelolipomas of the liver: A case report and literature review

**DOI:** 10.1097/MD.0000000000016497

**Published:** 2019-07-19

**Authors:** Huizhen Xin, Haijun Li, Honghui Yu, Juan Zhang, Weizhi Peng, Dechang Peng

**Affiliations:** Department of Radiology, The First Affiliated Hospital, Nanchang University, Jiangxi Province, The People's Republic of China.

**Keywords:** chemical shift imaging, hepatic, magnetic resonance imaging, myelolipoma

## Abstract

**Rationale:**

Primary hepatic myelolipoma is a rare benign neoplasm comprising mature adipose tissue and marrow components in various proportions. Chemical shift imaging (CSI) can distinguish the lipid within the tumor clearly; however, there have been no reports on the CSI of hepatic myelolipoma.

**Patient concern:**

A 20-year-old woman visited our hospital after discovering a space-occupying lesion in the liver with a history of more than 1 year. She felt distension pain and discomfort under the xiphoid process, accompanied by nausea, vomiting, and occasional chest oppression.

**Diagnosis:**

The tumor showed a well-defined mass with a pseudocapsule and a heterogeneous appearance on both T1- and T2-weighted magnetic resonance (MR) images. CSI analysis showed a signal decline within the tumor. Based on the histopathology, the tumor was diagnosed as hepatic myelolipoma.

**Interventions and Outcomes:**

The patient underwent a right hepatectomy, and the postoperative vital signs were stable. Two weeks later, the patient was discharged safely.

**Lessons:**

Although hepatic myelolipoma is extremely rare, this condition should be considered in differential diagnosis when CSI shows that hepatic lesions contain fatty.

## 1 Introduction

Myelolipoma is an unusual benign tumor, the majority of these tumors are located in the adrenal gland cortex, with approximately 0.4% detected as incidental findings in autopsies, and extra-adrenal occurrence being unusual.^[1]^ Myelolipoma in the liver is extremely rare; to date, only 11 cases have been reported in English language medical literature,^[2–12]^and only 1 case has reported magnetic resonance (MR) imaging of hepatic myelolipoma. Excellent soft tissue resolution is a feature of MR imaging, which is helpful for further characterizing the nature of the tumor. In the present study, the patient was the youngest to have a giant hepatic myelolipoma. We highlight the performance of MR imaging, especially chemical shift imaging (CSI), in the diagnosis of hepatic myelolipoma, which can increase our understanding of this tumor.

## 2 Case report

A 20-year-old woman visited our hospital due to the discovery of a space-occupying lesion in the liver with a history of more than 1 year. The patient underwent an upper abdominal computed tomography (CT) scan without enhancement because of experiencing occasional bouts of sphagitis a year ago. CT examination showed a giant low-density mass in the liver, and ultrasound revealed a hyperechoic focal lesion. The CT diagnosis by another hospital was angiomyolipoma. She felt distension pain and discomfort under the xiphoid process, which was a kind of intermittent pain with no radiation to other areas and was accompanied by nausea, vomiting, and occasional chest oppression. For further diagnosis and treatment, the patient came to our hospital. The abnormal laboratory results are as follows: alanine transferase 179 U/L (normal, 7–40), aspartic acid transpeptidase 78 U/L (normal,13–34), plasma total protein 54.5 g/L (normal, 65–85), albumen 39.1 g/L (normal, 40–55), globulin 15.4 g/L (normal, 20–40), ratio of albumin to globulin 2.54 (normal, 1.2–2.4), activation partial thromboplastin time 41.9 s (normal, 22.7–31.8), and fibrinogen 4.03 g/L (normal, 1.8–3.5).

**Figure 1:**
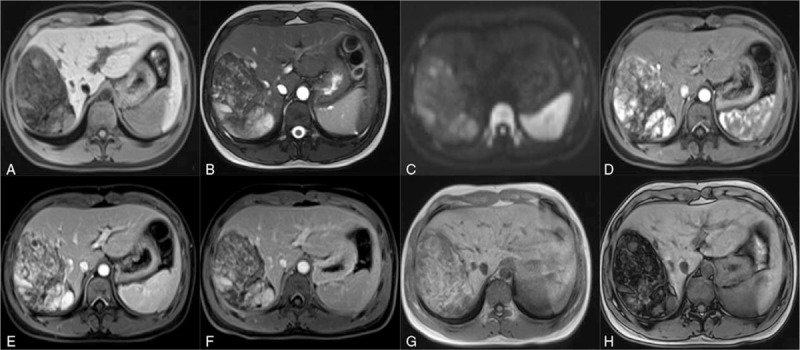
(A) Axial T1-weighted MRI shows a heterogeneous hypointense mass in the right lobe of the liver.(B) Axial T2-weighted MRI shows again the heterogeneous hyperintense mass with a clear boundary. (C) Diffusion-weighted imaging shows that the mass is hyperintense. (D) The arterial phase shows that the mass was initially obviously heterogeneously enhanced after the administration of gadolinium. (E) Both the portal phase and (F) delayed-phase images show that the enhancement degree of the lesion was decreased but still visible. (G)In-phase and (H) out-phase images show signal decline within the lesion. MRI = magnetic resonance imaging.

Upper abdominal MR imaging was carried out on a 1.5-T system (Siemens, Erlangen, Germany) using axial T1-weighted (T1W) turbo spin-echo (TSE), 2 plane T2W TSE, diffusion-weighted imaging sequences with large b value (b value = 800), and 2-dimensional T1Wdual echo in and opposed phase CSI, and gadolinium enhancement. MR imaging showed a heterogeneous T1 hypointense and T2 hyperintense giant mass of approximately 9.9^∗^9.1^∗^6.5 cm with a well-established capsule in the right lobe of the liver, which was mainly located in segment VII and presented hyperintensity on diffusion-weighted imaging. In addition, in-phase and out-phase images show a signal decline within the mass, which suggests that the lesion contains rich lipids. On dynamic contrast scanning, the lesion was initially obviously heterogeneous enhancement after the administration of gadolinium, and the enhancement degree of the lesion was decreased but still visible on both portal-phase and the delayed-phase images (Fig. 1).

**Figure 2:**
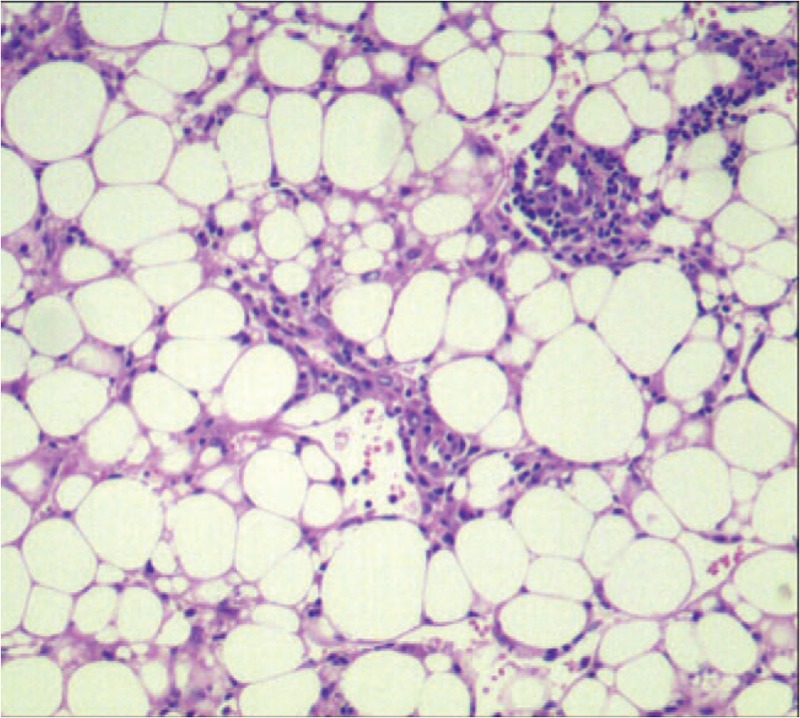
Microscopic examination revealed that the tumor consisted of mature adipose tissue and hematopoietic elements.(HE, 200 × ).

Clinically, the indication of the operation was clear because the patient was young and had a giant tumor, so a right hepatectomy was performed. The postoperative vital signs were stable, and the patient was discharged safely after 2 weeks. The lesion mainly occupied the right lobe of the liver and showed a clear boundary with the surrounding tissue. A right hepatectomy was carried out. The histological examination macroscopically revealed a 6.0^∗^4.5^∗^6.0 cm solid daffodil yellow tumor with hemorrhage and necrosis, and the surrounding liver tissue appeared greasy. Microscopically, the tumor comprising mature adipose tissue encapsulated by a thin fibrous septum and hematopoietic elements comprising 3 hematopoietic series, which consistent with myelolipoma (Fig. 2).

## 3 Discussion

Myelolipoma is a rare benign tumor of mesenchymal origin that consists of mature adipose tissue and marrow components in various proportions.^[13]^ The vast majority of myelolipomas occur in the adrenal gland, and extra-adrenal myelolipomas are not common. Currently, the documented sites include the pleura,^[14]^ lung,^[15]^ presacral area, ^[16]^ thoracic spine,^[17]^ right iliac fossa,^[18]^ mediastinum,^[19]^ and retroperitoneum perirenal tissue.^[14]^ Hepatic myelolipomas are extremely rare, with only 11 cases being well recorded in medical literature in the English language, including this case, and primary hepatic myelolipoma was reported in a total of 12 cases (Table 1). All of these cases were reported as individual cases. The average age of these 12 patients was 48.8 years (range 20–76 years), and the female-male ratio was 7:5. The occurrence of myelolipoma in the right liver lobe was reported in 9 cases (75%), and only 3 cases (25%) were reported in the left liver lobe. Most patients choose surgical resection for treatment.

**Table 1:**
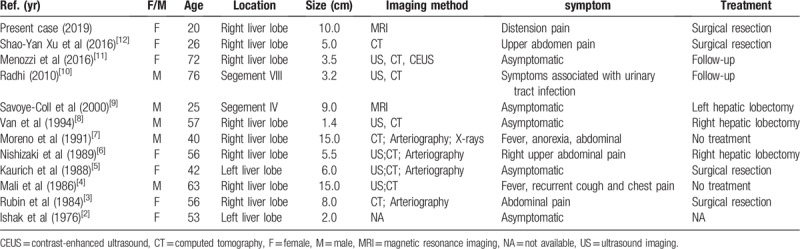
Clinic data of patients with hepatic myelolipoma in the English Medical Literature.

The etiology of hepatic myelolipoma is not clear, at present, speculations concerning the etiology include the ectopic adrenal gland and hepatocyte metaplasia.^[14,20]^ The adrenal gland is mainly the site of myelolipoma, and the right adrenal gland is closely related to the right lobe of the liver anatomically. Notably, most reported hepatic myelolipoma is located in the right lobe of the liver, which is further supported by this hypothesis. The liver is the primary hematological organ of the embryo and fetus until bone marrow begins to form. Therefore, myelolipoma as residual embryonic stem cells in liver tissue represents another hypothesis.

The discovery of hepatic myelolipoma by MR imaging has only been reported once before.^[9]^ In the previous case, the tumor showed a well-defined mass with a pseudocapsule and a heterogeneous appearance on both T1- and T2-weighted MR images, which is consistent with the present case. However, these researchers showed differences in enhanced images with gadolinium bolus. In our case, the tumor initially showed intense inhomogeneous enhancement and decreased enhancement degree with visibility on both portal-phase and delayed-phase images, while the former case demonstrated that the tumor was still hypointense in the arterial phase, and peripheral enhancement was observed on delayed-phase images, which is delayed heterogeneous enhancement. In addition, hepatic myelolipoma was reported to be homogeneously hyperenhanced in the arterial phase and slightly hyperechoic in the portal venous phase on contrast-enhanced ultrasound.^[11]^ This difference may be explained by the various proportions of fat and myelogenous components. Notably, compared to the former case, in our case, the lesion was subjected to additional kinds of MR imaging techniques, particularly CSI. The CSI technique is more accurate than frequency-selective fat suppression in identifying microscopic fat and is more sensitive because the former simply suppresses signals from fat so that a larger amount of fat is needed for its effect to be visible.^[21]^ The axial out-of-phase MR image displayed an intuitive signal decline in our case, which helped in further diagnosis and treatment.

Most hepatic myelolipomas were found incidentally and were usually not accompanied by clinical symptoms, except for the increasing tumor size or combined necrosis. In general, simple monitoring is enough for small and asymptomatic lesions, while surgical intervention is recommended for symptom ticlesions.

In conclusion, MRI, especially chemical shift imaging, helps to show the adipose tissue of the tumor and can identify microscopic lipids that can be neglected on CT scans. Although hepatic myelolipoma is a rare tumor, it should be considered in the differential diagnosis of space-occupied lesions in the liver. On MR imaging, the hepatic lesion showed a well-defined, pseudocapsule consisting of fatty tissue, indicating myelolipoma.

## Author contributions

**Conceptualization:** Huizhen Xin, Honghui Yu.

**Investigation:** Huizhen Xin, Haijun Li.

**Resources:** Honghui Yu, Juan Zhang, Zhipeng Wei.

**Writing – original draft:** Huizhen Xin.

**Writing – review & editing:** Dechang Peng, Haijun Li.
